# Zika virus infection induces ultrastructural alterations, mitochondrial dysfunction and oxidative stress in human trophoblast HTR-8/SVneo

**DOI:** 10.3389/fcimb.2026.1811377

**Published:** 2026-04-10

**Authors:** Laíza Vianna Arruda, Natália Gedeão Salomão, Sheila Maria Barbosa de Lima, Gisela Freitas Trindade, Luciana Lontro Alves, Fernanda Verdini Guimarães, Patrícia Machado Rodrigues Silva, Estela Bevilacqua, Daphne Pinheiro, Andre Luiz Mencalha, Silvio Rodrigues, Jemima Fuentes Ribeiro da Silva, Jorge José de Carvalho, Kíssila Rabelo

**Affiliations:** 1 Laboratory of Ultrastructure and Tissue Biology, Rio de Janeiro State University, Rio de Janeiro, Brazil; 2 Interdisciplinary Laboratory for Medical Research, Oswaldo Cruz Foundation, Rio de Janeiro, Brazil; 3 Laboratory of Virological Technology, Biomanguinhos, Oswaldo Cruz Foundation, Rio de Janeiro, Brazil; 4 Laboratory of Inflammation, Oswaldo Cruz Institute, Oswaldo Cruz Foundation, Rio de Janeiro, Brazil; 5 Laboratory for Studies on Trophoblast Biology and Maternal-Fetal Interaction, Institute of Biomedical Sciences, University of São Paulo, São Paulo, Brazil; 6 Cancer Biology Laboratory, Rio de Janeiro State University, Rio de Janeiro, Brazil; 7 Laboratory for Clinical and Experimental Research in Vascular Biology, Rio de Janeiro State University, Rio de Janeiro, Brazil

**Keywords:** arbovirus, host–pathogen interaction, maternal-fetal interface, mitochondria, placental infection

## Abstract

**Background:**

Zika fever gained importance in Brazil in 2015 due to its association with congenital syndrome. Although Zika virus (ZIKV) crosses the placenta and infects the fetus, its pathogenesis remains incompletely understood. This study investigated the effects of ZIKV infection in HTR-8/SVneo trophoblast cells.

**Methods:**

Cells were infected with ZIKV (MOI 0.1, 0.2, or 1) or Mock control for 24 or 48 hours. Infection rate and viability were assessed by immunofluorescence and flow cytometry. Ultrastructural changes were analyzed by transmission electron microscopy. Mitochondrial membrane potential was evaluated by flow cytometry. Gene expression related to mitochondrial dynamics, antioxidant response (*sod, cat, nrf2*), was analyzed by RT-qPCR. Protein expression (SOD, CAT, NRF2), enzymatic activities (SOD, CAT), and oxidative damage markers (8-OHdG, MDA, NO) were assessed by immunofluorescence and/or colorimetric assays.

**Results:**

MOI 1 for 24 hours produced the highest NS1 expression and infection rate (62.53%) and higher viability (89% vs. 28.1%), establishing this as the optimal condition. Infected cells exhibited mitochondrial damage, including ruptured membranes and loss of cristae, dilated endoplasmic reticulum, clusters of virus-like particles, and vesicle secretion. Mitochondrial membrane potential was reduced, along with decreased transcripts of genes involved in mitochondrial dynamics. Although *sod, cat*, and *nrf2* transcripts were reduced, protein immunolabeling and SOD activity were increased, whereas CAT activity was decreased. Elevated levels of 8-OHdG, MDA, and NO confirmed oxidative stress.

**Conclusion:**

ZIKV infection induces mitochondrial dysfunction, oxidative stress, and impaired mitophagy in HTR-8/SVneo trophoblast cells, highlighting mitochondrial dysfunction as a major component of the cellular response to ZIKV infection in trophoblast cells.

## Graphical Abstract

**Figure:**
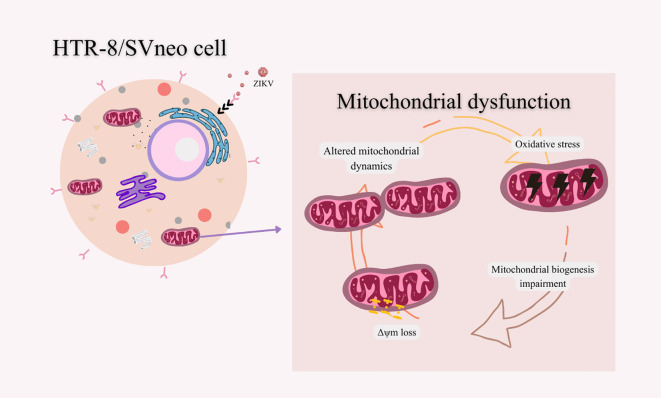
Infection of HTR-8/SVneo trophoblast cells by ZIKV is associated with mitochondrial dysfunction, characterized by altered mitochondrial dynamics, increased oxidative stress, loss of mitochondrial membrane potential (Δψm), and impaired mitochondrial biogenesis.

## 1 Introduction

Zika virus (ZIKV) is an arbovirus with a recognized potential to disproportionately affect maternal-fetal health due to its tropism for placental and neural tissues (Narasimhan et al., 2020). It was first detected in 1947 from the blood of *Rhesus* monkeys inhabiting the Zika forest in Uganda, Africa (Dick et al., 1952). However, its potential public health impact was not acknowledged until the first disease outbreaks in 2007 in Micronesia and in 2013 in French Polynesia (Dick et al., 1952). The virus gained prominence in Brazil in 2015, with cases initially reported in the Northeast region (Hills et al., 2017), which rapidly spread throughout the country. In 2016, the World Health Organization (WHO) declared the situation a public health emergency (Hills et al., 2017). Despite the decrease in reported cases since then, ZIKV continues to circulate in over 90 countries across Africa, the Americas, Europe, Southeast Asia, and the Western Pacific region (De and Grobusch, 2025).

ZIKV belongs to the *Orthoflavivirus* genus and shares the *Flaviviridae* family with other medically important arboviruses, including dengue virus and yellow fever virus (Rabe et al., 2025). It is a single-stranded, positive-sense RNA virus, enveloped, with icosahedral symmetry and a diameter of 25 to 60 nm (Rabe et al., 2025). Transmission occurs primarily through the bite of infected *Aedes* mosquitoes (*Stregomia* subgenus), but can also occur through sexual contact, blood transfusion, and vertical transmission (from mother to fetus) (Narasimhan et al., 2020).

Approximately 80% of ZIKV infections are asymptomatic or present with mild, self-limiting symptoms such as low-grade fever, arthralgia, myalgia, headache, fatigue, abdominal pain, lymphadenopathy, retro-orbital pain, conjunctivitis, and cutaneous rash (Rabe et al., 2025). However, ZIKV has also been associated with neurological complications, such as Guillain-Barré Syndrome (GBS), and gestational alterations related to Congenital Zika Syndrome (CZS) (Freitas et al., 2020; Filgueiras et al., 2021). CZS comprises a spectrum of congenital anomalies that include both structural malformations and functional impairments (Freitas et al., 2020). These manifestations encompass microcephaly, central nervous system abnormalities, ocular alterations, low birth weight, and neurodevelopmental delay (Freitas et al., 2020). It is estimated that up to 30% of infected pregnant women experience adverse fetal outcomes, including intrauterine growth restriction, fetal death, and central nervous system malformations (Wong et al., 2025).

Importantly, evidence indicates that fetal damage is not restricted to early gestation, as exposure during mid or late pregnancy can also lead to neuroinflammation and depletion of neural progenitor cells associated with persistent viral replication in placental and fetal tissues (Wong et al., 2025). Previous study of our group has demonstrated viral persistence throughout gestation in a prior study (Rabelo et al., 2020), reinforcing that sustained ZIKV replication may contribute to ongoing fetal damage. These observations highlight the importance of understanding how ZIKV interacts with placental cells and disrupts the maternal–fetal interface.

The human placenta is a highly specialized organ responsible for nutrient exchange and fetal protection during pregnancy. Structurally, it is composed of chorionic villi lined by trophoblast cells that form the primary interface between maternal and fetal tissues Kojima et al., 2022; Alves et al., 2023). The outer multinucleated syncytiotrophoblast layer is directly exposed to maternal blood, whereas the underlying cytotrophoblasts can differentiate into extravillous trophoblasts that invade the maternal decidua and remodel uterine spiral arteries to establish adequate placental perfusion (Vornic et al., 2025). Because of their invasive phenotype and direct interaction with maternal tissues, extravillous trophoblasts represent a potential entry point for pathogens reaching the placenta through maternal circulation (Zeldovich et al., 2011). Consistent with this susceptibility, ZIKV has been shown to infect several placental cell types, including trophoblast populations, enabling viral replication and facilitating vertical transmission to the fetus (Tabata et al., 2016). In addition to trophoblasts, endothelial cells such as human umbilical vein endothelial cells (HUVECs) have also been reported to be permissive to ZIKV infection, highlighting the broad cellular susceptibility within the maternal–fetal interface (Cheng et al., 2018).

To better understand ZIKV infection mechanisms at the maternal–fetal interface, different trophoblast−derived cell lines have been used as experimental models. Among them, the choriocarcinoma−derived cell lines BeWo and JEG−3 have been extensively employed to study host responses to infection in placental trophoblasts; both cell types support infection and replication by ZIKV, although primary human trophoblasts can be relatively resistant *in vitro* (Morosky et al., 2016). In these models, infection triggers antiviral signaling pathways characterized by type I and type III interferon responses and the induction of antiviral interferon−stimulated genes (ISGs) such as ISG15, IFIT1, and MX1 (observed in ZIKV−infected placental cells and explants), indicating that trophoblasts mount innate immune responses despite supporting replication (Van der Hoek et al., 2025). Despite this antiviral response, ZIKV remains capable of replicating in trophoblast−derived cells while inducing cellular stress and apoptosis, supporting their use to study the balance between host defense and viral replication in placental tissue (Muthuraj et al., 2021).

Comparative transcriptomic analyses in trophoblast-derived models show that ZIKV infection induces broad transcriptional and metabolic alterations. In BeWo cells, infection alters genes related to lipid metabolism and placental transport, suggesting impacts on maternal–fetal nutrient exchange (Wu et al., 2023). In JEG-3 cells, transcriptomic profiling reveals changes in gene expression, including activation of immune and inflammatory pathways and upregulation of genes associated with endoplasmic reticulum stress, highlighting the complex cellular stress responses triggered by infection (Chen et al., 2022; Cervantes et al., 2023; Velazquez-Cervantes et al., 2024).

In addition to choriocarcinoma-derived trophoblast models, the HTR-8/SVneo cell line has been widely used to investigate placental physiology and host–pathogen interactions at the maternal–fetal interface. This cell line was originally derived from first-trimester human extravillous trophoblasts and retains key features of invasive trophoblasts, including the capacity for migration and interaction with maternal decidual tissues (Graham et al., 1993; Abou-Kheir et al., 2017). Because extravillous trophoblasts play a critical role in placental implantation and remodeling of maternal spiral arteries, they represent an important cellular population exposed to pathogens circulating in maternal blood. Therefore, HTR-8/SVneo cells provide a relevant experimental model to investigate mechanisms of viral infection and cellular responses in trophoblasts with invasive properties at the maternal–fetal interface.

ZIKV exhibits broad cellular tropism at the maternal-fetal interface, infecting cytotrophoblasts cells, decidual cells, fetal endothelial cells, and Hofbauer cells (Tabata et al., 2016). Infection disrupts placental homeostasis, impairing trophoblast functions through multiple cellular mechanisms (Wong et al., 2025). Several studies have shown that ZIKV infection leads to the accumulation of reactive oxygen species (ROS) and persistent endoplasmic reticulum stress in the trophoblast, resulting in apoptotic pathway activation and contributing to placental damage (Pereira et al., 2021; Lebeau et al., 2022). Beyond its impact on the endoplasmic reticulum, accumulating evidence indicates that ZIKV also interferes with mitochondrial function and dynamics, leading to alterations in cellular metabolism and redox balance (Wang et al., 2013; Figarola-Centurion et al., 2023). However, despite the central role of mitochondria in regulating trophoblast metabolism and survival, studies specifically addressing the effects of ZIKV infection on mitochondrial dynamics in trophoblast cells remain scarce.

Investigating the effects of ZIKV infection on this organelle is highly relevant, considering that mitochondrial dysfunction has been associated with placental dysfunction, reducing trophoblast invasiveness, and consequently impairing spiral artery remodeling (Hu and Zhang, 2021). Moreover, mitochondrial dysfunction has been linked to adverse pregnancy outcomes, including pre-eclampsia and intrauterine growth restriction (Yildirim et al., 2022), suggesting it may be a key component in understanding ZIKV pathogenesis.

Accordingly, the present study aimed to characterize the impacts of ZIKV infection in trophoblast cells *in vitro*, evaluating morphological alterations by ultrastructural analysis, as well as potential changes in mitochondrial function and dynamics and oxidative stress. This approach is essential to understand the cellular mechanisms that contribute to the maternal-fetal pathogenesis of the virus.

## 2 Materials and methods

### 2.1 Cell culture and virus

HTR-8/SVneo cells (human first-trimester extravillous trophoblast cell line; ATCC) were maintained at 37 °C in a humidified atmosphere containing 5% CO_2_, in RPMI-1640 medium (Sigma-Aldrich, USA) supplemented with 10% fetal bovine serum (FBS, Cultilab, Brazil) and penicillin/streptomycin (100 U/mL, Sigma-Aldrich, USA). The virus used in this work was the ZIKV PB81, originally isolated from a patient in Paraíba, Brazil, propagated in C6/36 cells and titrated in Vero cells (CCL-81), resulting in a viral titer of 5.8 × 10^6^ PFU/mL.

### 2.2 Standardization of HTR-8/SVneo cells infection

To optimize ZIKV infection in HTR-8/SVneo trophoblast cells, viral inoculation was performed at multiplicities of infection (MOI) 0.1, 0.2, and 1.0. Cells were exposed to the virus in a serum-free medium for 1 h at 37 °C. After incubation, the inoculum was removed, and cells were maintained in RPMI-1640 (Sigma-Aldrich, USA) medium supplemented with 10% of fetal bovine serum (FBS) (Cultilab, Brazil) for 24 or 48 h. MOCK-infected controls, consisting of equivalent volumes of supernatant from uninfected Vero cells, were included in all experiments. Infection efficiency was evaluated by flow cytometry and immunofluorescence, and the optimal infection kinetics were determined using a cell viability assay with trypan blue (Sigma-Aldrich, USA) exclusion.

### 2.3 Assessment of infection rate by flow cytometry

Cells were trypsinized, fixed in 4% paraformaldehyde, permeabilized with 0.25% Triton, blocked with 3% bovine serum albumin (BSA), and stained with anti-NS1 primary antibody (1:1000, ARG65781, Arigo Biolaboratories, Taiwan) and Alexa 488-conjugated secondary anti-mouse antibody (1:500, A-11001, Thermo Fisher Sci. Inc., USA). Analysis was performed using a CytoFlex cytometer and Summit 6.1 software (Beckman Coulter, USA).

### 2.4 Immunofluorescence for infection standardization

For NS1 protein detection, assessment of mitochondrial quality and antioxidant responses, cells were fixed with 4% paraformaldehyde for 15 min at room temperature. Cells were then permeabilized with 0.6% saponin containing 1% BSA for 20 min at room temperature, followed by blocking with 2% BSA for 30 min at the same temperature Samples were incubated overnight at 4 °C with the following primary antibodies: mouse monoclonal anti-NS1 (1:300, ARG65781, Arigo Biolaboratories), mouse monoclonal anti-PGC-1α (1:100, Sc:518025, Santa Cruz Biotechnology), rabbit polyclonal anti-SOD (1:100, PA1345, BosterBio), rabbit polyclonal anti-CAT (1:100, PB9925, BosterBio), rabbit monoclonal anti-NRF2 (1:100, E5F1A, Cell Signaling Technology), and goat polyclonal anti-8-OHdG (1:100, NB600-150, Novus Biologicals). Subsequently, cells were incubated with Alexa 488- or Alexa 555-conjugated anti-mouse, anti-rabbit, or anti-goat secondary antibodies (1:500, Thermo Fisher Scientific) for 1 h at room temperature. Slides were mounted with Fluoroshield containing DAPI (F6057, Thermo Fisher Scientific) and analyzed by confocal microscopy (Leica TCS SP8 MP). Fluorescence intensity was quantified using ImageJ software (version 1.54k; NIH, USA).

### 2.5 Ultrastructural analysis by transmission electron microscopy

Cells were trypsinized and fixed with 2.5% glutaraldehyde in sodium cacodylate buffer (0.1 M, pH 7.2), post-fixed with 1% buffered osmium tetroxide, dehydrated in an acetone series (30, 50, 70, 90, and 100%), and embedded in EPON 812 (Electron Microscopy Sciences, USA) polymerized at 60 °C for 3 days. Ultrathin sections (60–90 nm) were contrasted with uranyl acetate and lead citrate and were visualized using a JEOL 1001 transmission electron microscope (Jeol Ltd., Japan).

### 2.6 Mitochondrial membrane potential assay (ΔΨm)

Mitochondrial membrane potential was evaluated in HTR-8/SVneo cells infected with Zika virus at an MOI 1 or MOCK-treated for 24 h using JC-10 dye (Mitochondrial Membrane Potential Assay Kit, Sigma-Aldrich, USA), according to the manufacturer’s instructions. Carbonyl cyanide 3-chlorophenylhydrazone (CCCP, 10 μM for 30 min) was used as a positive control for mitochondrial depolarization. Fluorescence intensity was quantified by flow cytometry (CytoFLEX S, Beckman Coulter). JC-10 fluorescence was detected in the green (monomeric) and red (aggregate) channels. Mitochondrial depolarization was quantified as the Green/Red fluorescence ratio, representing the relative abundance of monomeric versus aggregated dye, and analyzed using ImageJ software.

### 2.7 RNA isolation and quantitative RT-PCR

RNA was extracted from HTR-8/SVneo cells infected with Zika virus MOI 1 or MOCK-treated for 24 h using TRIzol reagent (Invitrogen, USA). After phase separation with chloroform and centrifugation at 13,000 rpm for 15 min at 4 °C, the aqueous phase was collected, and RNA was precipitated with isopropanol, washed, and resuspended in DEPC-treated water. RNA concentration and purity were determined using a NanoDrop 1000 spectrophotometer (Thermo Fisher Sci. Inc, USA), and samples were stored at –70 °C. Residual genomic DNA was removed with DNase I (Invitrogen, USA) according to the manufacturer’s instructions. For cDNA synthesis, 2 μg of DNase-treated RNA were reverse-transcribed using the GoScript Reverse Transcriptase cDNA Synthesis Kit (Promega, USA) with Oligo(dT) primers and Superscript IV reverse transcriptase (Thermo Fisher Sci. Inc, USA). Quantitative real-time PCR (RT-qPCR) was performed using diluted cDNA (1:5) and Rotor-Gene SYBR Green PCR Master Mix (Qiagen, Germany) in a Rotor-Gene 6000 thermocycler. Amplification reactions (10 μL) contained 0.5 μM of each primer targeting *mff, fis1, mfn1, mfn2, dmnl1, opa1, pgc1α, nrf2, cat, or sod*. Gene expression levels were normalized to β-actin (ACTB), and relative expression was calculated using the ΔΔCt method. All reactions were performed in triplicate. The following RT-qPCR primers were used: *MFF* fw – TCAACTCCCTTTAAACCCCTG, rev -TTTCATCTCAAGACCGCTCTCT; *FIS 1* fw – TTTGAGTACGCCTGGTGCCT, rev – ACGGCCAGGTAGAAGACGTA; *MFN1* fw – GCAGTGGGAAGAGCTCTGT, rev – GCAGTGGGAAGAGCTCTGT; *MFN2* fw -GCAGTGGGAAGAGCTCTGT, rev – TTGATCACGGTGCTCTTCCC; *DMNL1* fw – AGGATCATTCAAGCACTGTAG, rev – CCAGTTCAATTGCCACTAAG; *OPA1* fw – TGGTGCTTGTTGACCTACCA; rev-CGTTCAGCATCCACAGATC; *PGC1A* fw- GGACTCAAGTGGTGCAGTGA. Rev – GGCGATCAAATGAGGGCAAT; *NRF2* fw – GGCGATCAAATGAGGGCAAT; rev – GGCGATCAAATGAGGGCAAT; *CAT* fw – TAAGACTGACCGAGGGCAT, rev – TCAAACCTTGGTGAGATC; *SOD* fw – ACAGGCCTTATTCACCTGCT, rev – CAGCATAACGATCGTGGTTG.

### 2.8 Evaluation of oxidative stress biomarkers

Cells infected with Zika virus at an MOI 1 or MOCK-treated for 24 h were homogenized in 200 μL of potassium phosphate buffer containing EDTA (KPE, pH 7.5) and centrifuged at 4,000 rpm for 10 min at 4 °C. The supernatant was collected, and samples were stored at –80 °C until biochemical analyses were performed. Nitric oxide (NO) levels were determined using the Griess reagent (Sigma-Aldrich, USA). Briefly, 100 μL of each sample were mixed with 100 μL of Griess reagent and incubated for 10 min protected from light. Absorbance was measured at 540 nm (SpectraMax M5, Molecular Devices, USA), and results were expressed as μmol/mg protein. Lipid peroxidation was assessed by quantifying malondialdehyde (MDA) using the thiobarbituric acid reactive substances (TBARS) method. Cell lysates (100 μL) were mixed with 100 μL of 10% trichloroacetic acid, centrifuged (3,600 × g, 15 min, 4 °C), and the supernatant was incubated with 150 μL of thiobarbituric acid (TBA) at 95 °C for 10 min. Absorbance was measured at 532 nm (SpectraMax M5, Molecular Devices, USA), and MDA levels were expressed as nM/mg protein.

### 2.9 Determination of antioxidant enzyme activity

For the determination of enzymatic antioxidant activity were processed as described above. Superoxide dismutase (SOD) activity was assessed by monitoring the inhibition of epinephrine auto-oxidation in glycine buffer (pH 10.2). The reaction mixture consisted of 1–3 μL of sample, 193 μL of glycine buffer, 2 μL of catalase, and 4 μL of epinephrine, with absorbance measured immediately at 480 nm using a spectrophotometer (SpectraMax M5, Molecular Devices, USA). Catalase (CAT) activity was determined by measuring the rate of hydrogen peroxide (H_2_O_2_) decomposition. Reactions were prepared by adding 1 μL of sample to 99 μL of a mix containing 25 mL of distilled water and 40 μL of H_2_O_2_. The decrease in absorbance at 240 nm during the first minute of reaction was recorded using a spectrophotometer (SpectraMax M5, Molecular Devices, USA).

### 2.10 Statistical analysis

All data were analyzed using GraphPad Prism software version 8.0 (GraphPad Software, USA) with the non-parametric Mann–Whitney test. For all assays, results shown in the graphs represent the mean ± standard deviation of three independent experiments performed in triplicate. Differences between samples were considered statistically significant when P < 0.05.

## 3 Results

### 3.1 Standardization of HTR-8/SVneo cells infection

**Figure 1:**
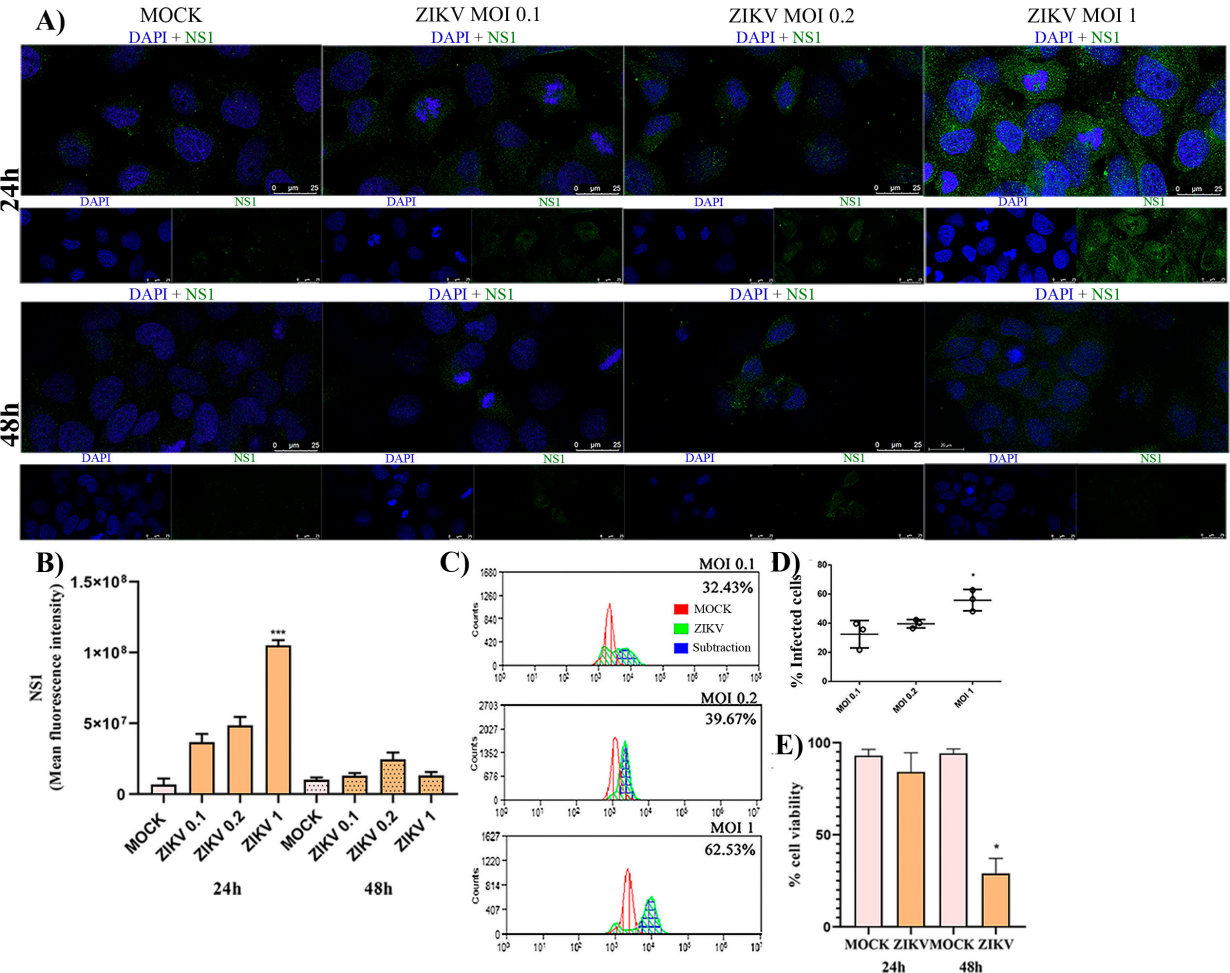
**(a)** Immunofluorescence micrographs showing NS1 protein expression at 24- and 48-hours post-infection (hpi) with MOI 0.1, 0.2, or 1 of ZIKV. **(b)** Quantification of fluorescence intensity revealed the highest NS1 signal at MOI 1. **(c)** Representative flow cytometry histograms showing the percentage of NS1-positive HTR-8/SVneo cells after infection with increasing MOIs. The maximal infection rate was observed at MOI 1, with 62.53% of NS1-expressing cells. **(d)** Mean percentage (± SD) of NS1-positive cells from three independent experiments. **(e)** Cell viability assay showing higher viability at 24 hpi (84%) compared with 48 hpi (29%). MOCK-infected cells served as negative control. Statistical analysis was performed using the Mann–Whitney test (*p < 0.05; ***p < 0.0001).

To establish a standardized infection protocol, HTR-8/SVneo cells were infected with Zika virus (ZIKV) at different multiplicities of infection (MOIs; 0.1, 0.2, and 1) for 24 or 48 h. ZIKV NS1 protein expression was evaluated by immunofluorescence and flow cytometry, and cell viability was assessed using the trypan blue exclusion assay. Immunofluorescence analysis revealed ZIKV replication in HTR-8/SVneo cells at all tested MOIs (**Figure 1A**). The highest NS1 fluorescence intensity was observed at MOI 1 after 24 h post-infection (hpi) (**Figure 1B**). Flow cytometry confirmed these findings in three independent experiments, showing that MOI 1 resulted in the highest proportion of NS1-positive cells (62.53%), compared with 32.43% and 39.67% for MOI 0.1 and 0.2, respectively (**Figures 1C, D**). Cell viability was significantly higher after 24 h of infection (84%) compared with 48 h (29%) (**Figure 1E**). These results indicate that an MOI 1 for 24 hours provides optimal conditions for ZIKV infection in HTR-8/SVneo cells, ensuring efficient viral replication with minimal cytotoxicity. All subsequent experiments were performed under these standardized conditions.

### 3.2 Ultrastructural alterations in ZIKV-infected trophoblast cells

**Figure 2:**
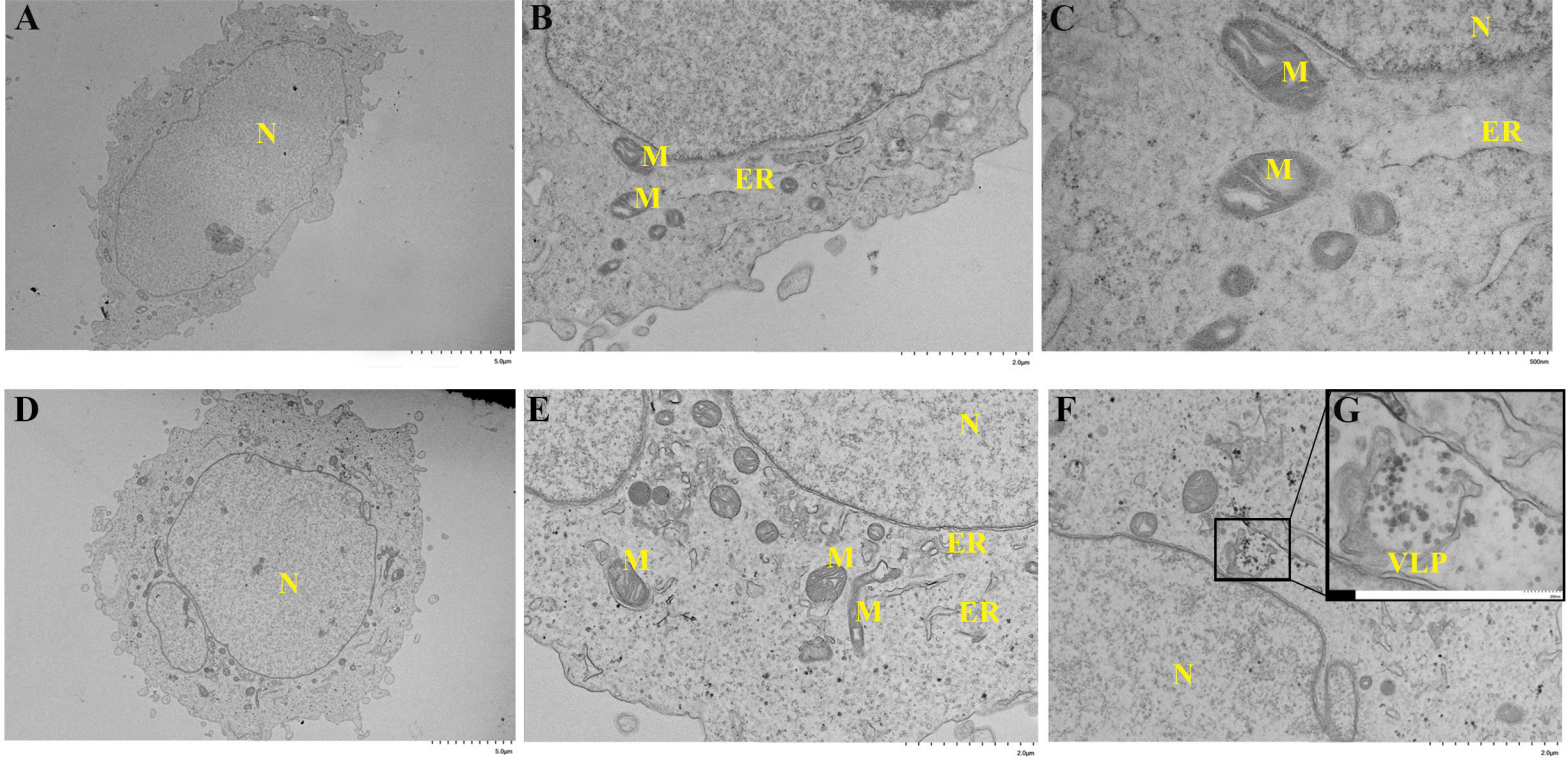
Ultrastructural analysis of HTR-8/SVneo cells infected with ZIKV. **(a–c)** MOCK-infected cells showing normal ultrastructure, with few organelles, intact nuclei and mitochondria, and endoplasmic reticulum (ER) with minimally dilated cisternae. **(d–g)** ZIKV-infected cells display a rounded morphology with cytoplasmic extensions and an increased density of organelles. Mitochondria exhibit structural damage, including ruptured membranes and loss of cristae (arrow). The ER appears more abundant and dilated, and clusters of virus-like particles (VLPs) consistent with ZIKV size are observed. Mitochondria (M), endoplasmic reticulum (ER), nucleus (N), and virus-like particles (VLP). Scale bars: **(a,d)** 2 μm; **(b,c,e,f)** 500 nm; **(g)** 200 nm.

Transmission electron microscopy (TEM) of MOCK-treated HTR-8/SVneo cells demonstrated well-preserved ultrastructural features, with intact nuclei and mitochondria, as well as an endoplasmic reticulum (ER) with only occasional cisternal dilatations, consistent with the high protein synthesis activity expected in trophoblast cells (**Figures 2A–C**). In contrast, ZIKV-infected cells displayed distinct ultrastructural alterations, including membrane extensions typical of activated cells, increased vesicle secretion, and a notably expanded ER with extensively dilated cisternae. Clusters of virus-like particles (VLPs) were also observed, corroborating the susceptibility and permissiveness of trophoblast cells to ZIKV infection. Additionally, infected cells exhibited mitochondrial alterations, including disrupted membranes and loss of cristae, indicative of infection-induced mitochondrial damage (**Figures 2D–F**).

### 3.3 ZIKV infection induces loss of mitochondrial membrane potential in trophoblast cells

Given the mitochondrial alterations observed by ultrastructural analysis, the mitochondrial membrane potential (ΔΨm) was further assessed in infected HTR-8/SVneo cells using the JC-10 probe. In healthy mitochondria, JC-10 aggregates within the matrix, emitting red fluorescence, whereas under stress conditions the loss of ΔΨm prevents aggregation, resulting in increased monomeric green fluorescence. CCCP, an oxidative phosphorylation uncoupler, was used as a positive control for mitochondrial depolarization with the shortest incubation time recommended by the manufacturer.

**Figure 3:**
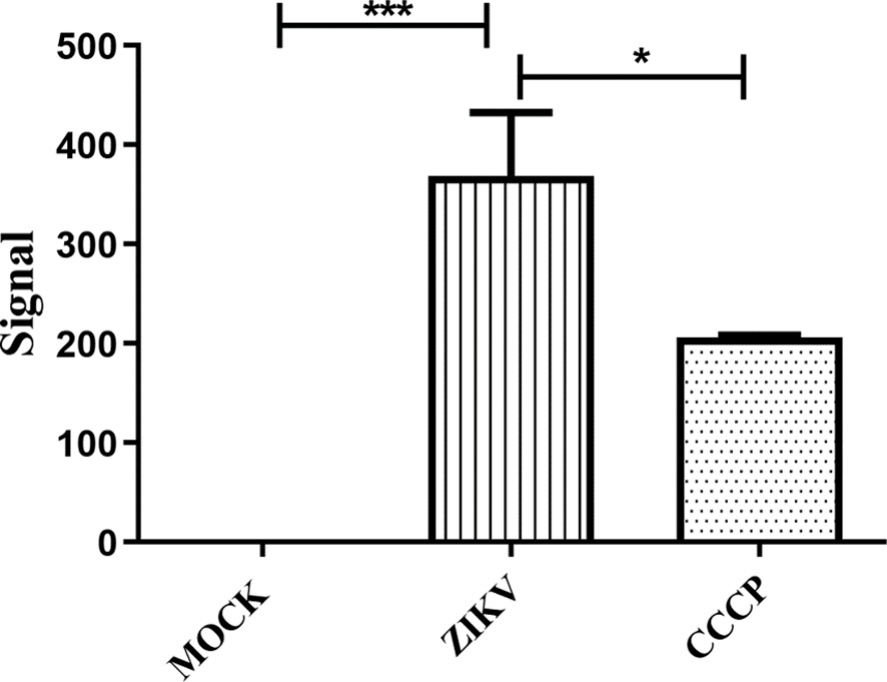
Mitochondrial membrane potential analysis in ZIKV-infected HTR-8/SVneo cells. **(a)** ZIKV-infected cells exhibited strong green fluorescence intensity, exceeding that of the positive control (CCCP), indicating mitochondrial membrane potential loss. **(b)** Representative histograms from three independent experiments showing fluorescence intensity in MOCK, ZIKV-infected, and CCCP-treated cells. Statistical analysis was performed using the Mann–Whitney test (*p < 0.05; ***p < 0.0001).

MOCK-infected cells displayed low green fluorescence intensity and a near-baseline green/red ratio (0.025± 0.608), indicating preserved mitochondrial potential. In contrast, ZIKV-infected cells exhibited strong green JC-10 fluorescence, and flow cytometry histograms revealed a clear shift of the cell population toward higher green fluorescence. Quantification of mitochondrial depolarization, expressed as the Green/Red fluorescence ratio (monomers/aggregates), showed a marked increase in infected cells (368.6 ± 63.75) compared with MOCK. These values were derived from the mean fluorescence intensity of the green and red channels obtained by flow cytometry. CCCP-treated cells also showed increased depolarization (1.942 ± 206.2), confirming the sensitivity of the assay. (**Figure 3**).

### 3.4 ZIKV infection disrupts mitochondrial dynamics in trophoblast cells

**Figure 4:**
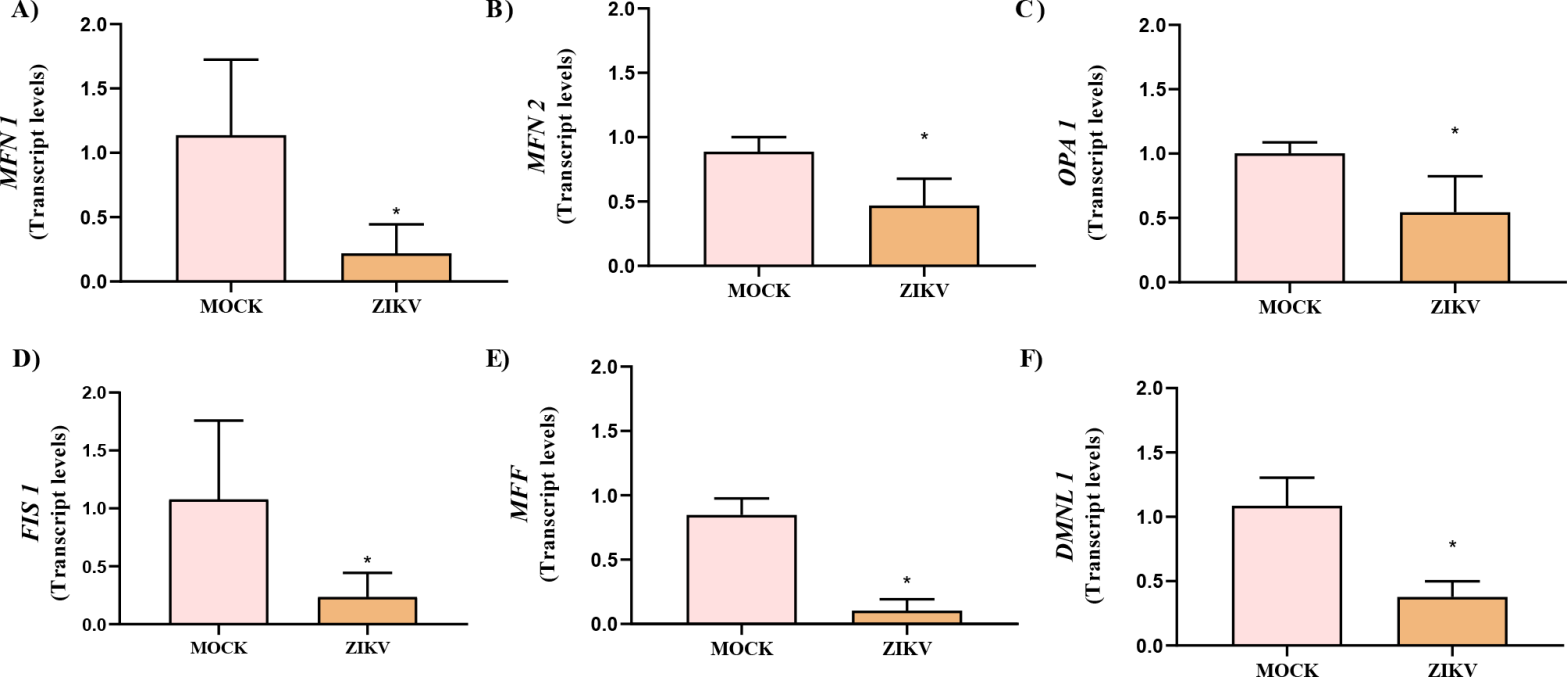
Gene expression of mitochondrial dynamics regulators in ZIKV-infected cells. **(a–f)** Relative mRNA levels of *MFN1*, *MFN2*, *OPA1*, *FIS1*, *MFF*, and *DNML1* in MOCK and ZIKV-infected HTR-8/SVneo cells. ZIKV infection significantly reduced the expression of both fusion-related (*MFN1*, *MFN2*, *OPA1*) and fission-related (*FIS1*, *MFF*, *DLMN1*) genes, suggesting disrupted mitochondrial dynamics. Statistical significance was determined using the Mann–Whitney test (*p < 0.05).

Given the mitochondrial damage previously observed, we analyzed the expression of genes related to mitochondrial dynamics by RT-qPCR to evaluate the integrity of mitochondrial quality control mechanisms during ZIKV infection. All seven analyzed genes: *MFN1, MFN2, OPA1, FIS1, MFF, DNML1*, and *PGC-1A*, were differentially expressed between ZIKV-infected (ZIKV) and control (MOCK) cells. A marked reduction in transcript levels was detected in both, fusion-related genes: *MFN1* (0.2 ± 0.2 *vs*. 1.1 ± 0.58), *MFN2* (0.5 ± 0.2 *vs*. 0.9 ± 0.1), and *OPA1* (0.5 ± 0.2 *vs*. 1.0 ± 0.08), and fission-related genes: *FIS1* (0.2 ± 0.2 *vs*. 1.0 ± 0.7), *MFF* (0.1 ± 0.08 *vs*. 0.8 ± 0.1), and *DNML1* (0.3 ± 0.1 *vs*. 1.0 ± 0.2), in infected cells compared with control, respectively (**Figures 4A–F**). Overall, ZIKV infection led to a reduction exceeding 40% in the transcript levels of genes involved in mitochondrial dynamics, suggesting a coordinated suppression of both mitochondrial fusion and fission pathways during viral infection.

**Figure 5:**
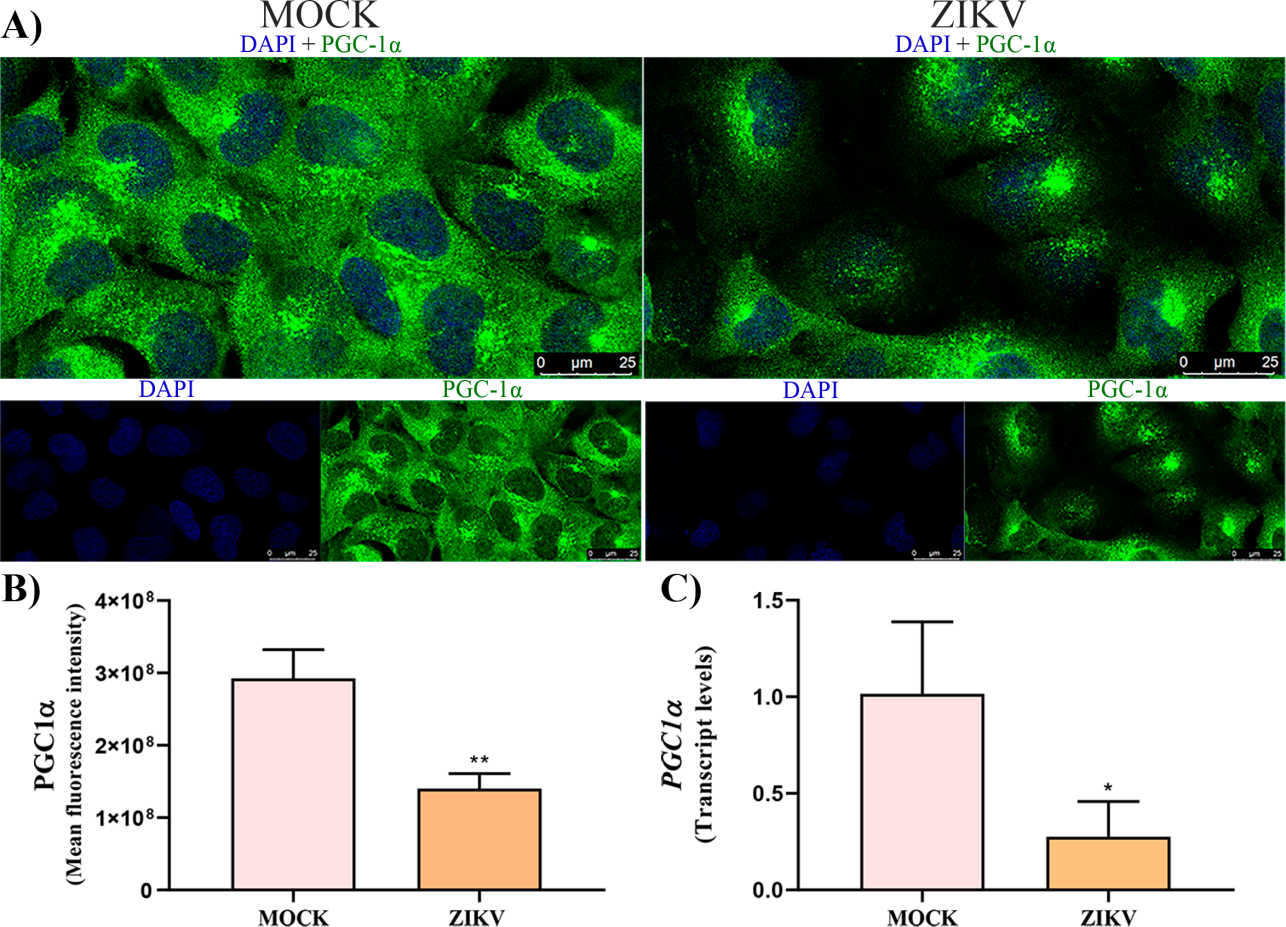
PGC-1α expression in ZIKV-infected HTR-8/SVneo cells. **(a)** Representative immunofluorescence micrograph of PGC-1α expression. **(b)** Quantification of fluorescence intensity showing reduced PGC-1α levels in ZIKV-infected HTR-8/Svneo cells compared with MOCK -infected cells. **(c)** RT-qPCR analysis confirming decreased *pgc-1α* mRNA expression in infected cells. Statistical significance was determined using the Mann–Whitney test (*p < 0.05; **p < 0.01).

Regarding *PGC-1A*, a significant decrease in transcript levels was observed in ZIKV-infected cells (0.3 ± 0.2) compared with control (1 ± 0.3) (**Figure 5C**). Consistently, immunofluorescence analysis demonstrated a pronounced reduction in PGC-1α protein signal intensity in infected cells (1.4 × 10^9^ ± 2.0 × 10^9^) relative to the control (2.9 × 10^9^ ± 3.9 × 10^9^) (**Figures 5A, B**), indicating a substantial downregulation of PGC-1α at both the transcriptional and protein levels during ZIKV infection.

### 3.5 ZIKV infection modulates cellular antioxidant defense mechanism

**Figure 6:**
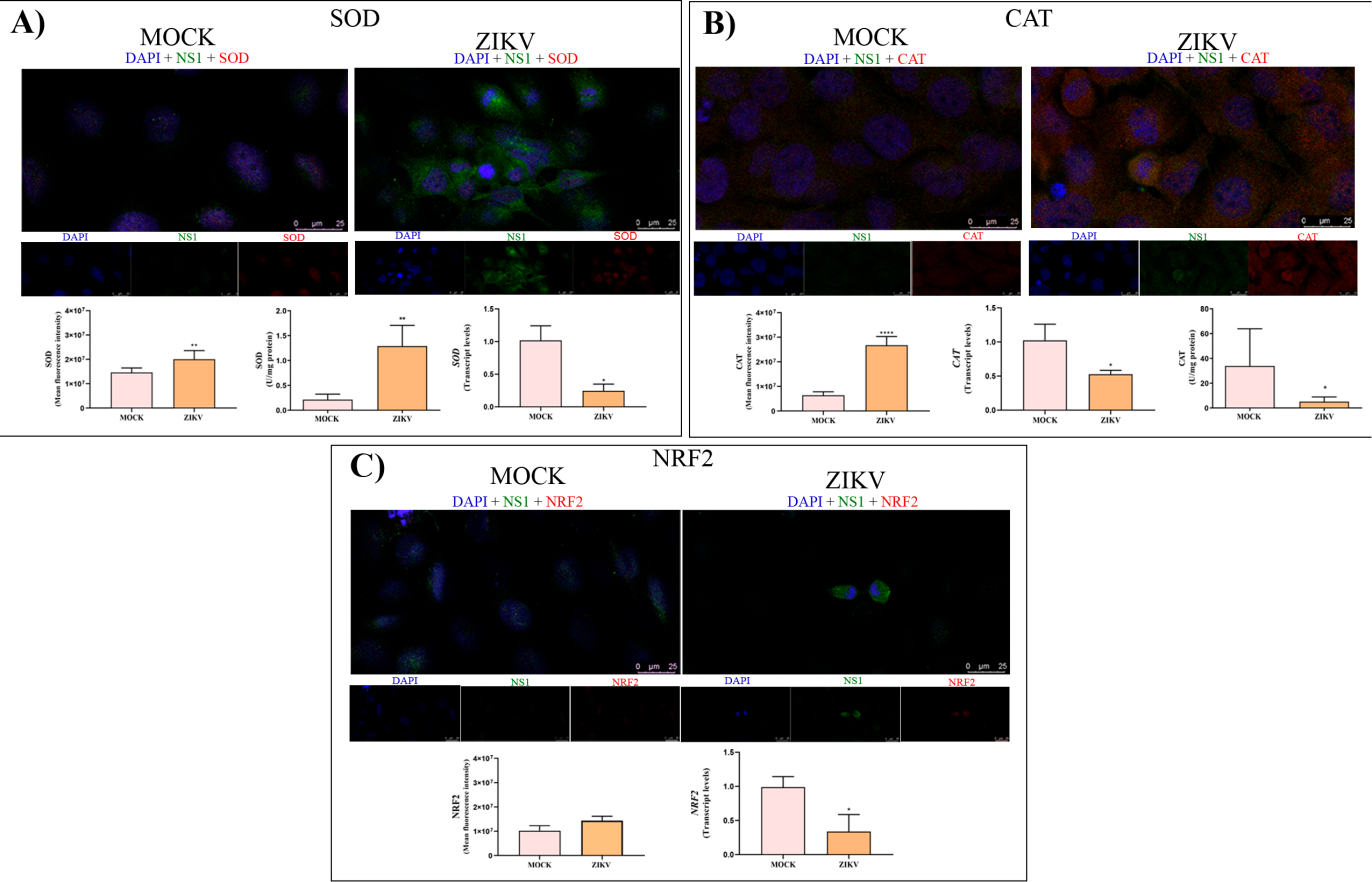
Expression of antioxidant response proteins in ZIKV-infected HTR-8/SVneo cells. **(a)** SOD analysis showing representative immunofluorescence images of ZIKV NS1 co-localization with SOD, fluorescence intensity quantification, gene expression levels, and enzymatic activity in ZIKV-infected cells compared with MOCK. **(b)** CAT analysis showing immunofluorescence images of NS1 co-localization with CAT, fluorescence intensity quantification, gene expression analysis, and enzymatic activity after infection. **(c)** NRF2 analysis showing immunofluorescence images of NS1 co-localization with NRF2, fluorescence intensity quantification, and gene expression levels in infected cells. Statistical significance was determined using the Mann–Whitney test (*p < 0.05; **p < 0.01; ****p < 0.0001).

Based on previous findings, we investigated potential alterations in the expression and activity of key enzymes involved in the cellular antioxidant response. In ZIKV-infected cells, SOD protein levels, as inferred from immunofluorescence intensity, were significantly increased (1.4×10^8^ ± 1.8 *vs*. 2.0×10^8^ ± 3.5 A.U., respectively), accompanied by a pronounced increase in enzymatic activity compared to controls (1.29 ± 0.41 *vs*. 0.21 ± 0.11 U/mg protein, respectively) (**Figure 6A**). Interestingly, despite the enhanced protein signal and activity, *SOD* transcript levels were markedly reduced in infected cells (0.2 ± 0.1 *vs*. 1.0 ± 0.2 A.U.) (**Figure 6A**). Likewise, immunofluorescence quantification revealed that CAT protein levels were markedly higher in infected cells than in controls (2.6×10^8^ ± 3.5 *vs*.). 6.3×10^8^ ± 1.4 A.U.) (**Figure 6B**). However, in contrast to protein abundance, CAT enzymatic activity was significantly reduced in infected cells relative to controls (5.1 ± 3.8 *vs*. 33.88 ± 30.0 U/mg protein, respectively) (**Figure 6B**). Concordantly, *CAT* transcript levels were also significantly decreased in infected cells compared to control (0.5 ± 0.05 *vs*. 1.0 ± 0.2 A.U.) (**Figure 6B**), suggest that ZIKV infection induces a complex regulation of antioxidant enzymes, involving both transcriptional suppression and potential post-transcriptional or compensatory regulatory mechanisms.

Additionally, a significant decrease in *NRF2* transcript levels was observed in ZIKV-infected cells compared to controls (0.3 ± 0.2 *vs*. 1.0 ± 0.1 A.U.) (**Figure 6C**). Although not statistically significant, a trend toward increased NRF2 protein levels was detected in infected cells (1.4 ± 1.7 × 10^8^ A.U.) relative to controls (1.0 ± 2.1 × 10^8^ A.U.), as determined by immunofluorescence intensity (**Figure 6C**).

### 3.6 ZIKV infection induces oxidative DNA damage and lipid peroxidation

**Figure 7:**
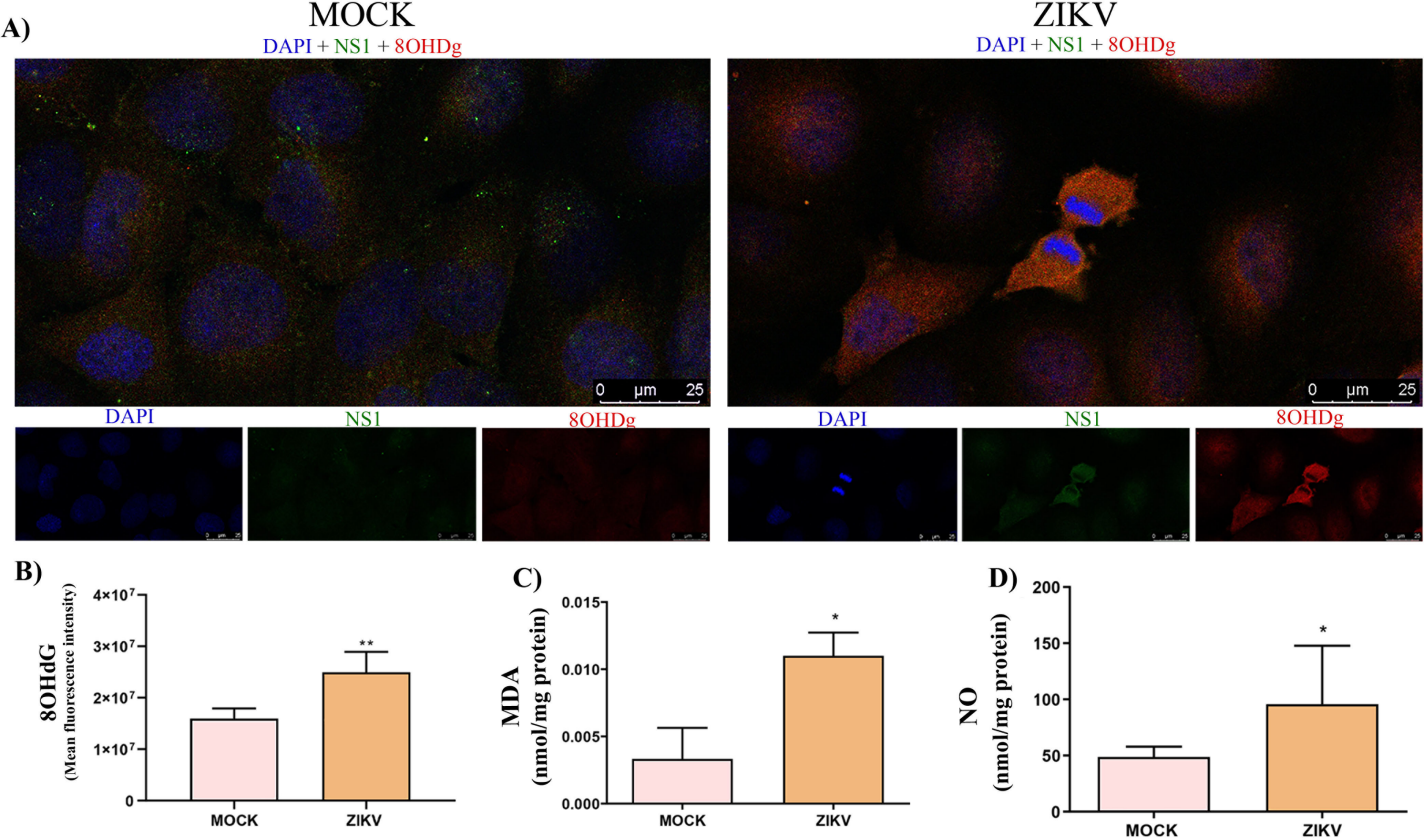
Biochemical markers of oxidative stress in ZIKV-infected HTR-8/SVneo cells. **(a)** Immunofluorescence micrographs showing 8-OHdG expression in MOCK and ZIKV-infected cells. **(b)** Quantification of fluorescence intensity showing accumulation of 8-OHdG in infected cells, indicating DNA oxidative damage. **(c, d)** Quantification of MDA and NO levels showing significant increases in both markers in infected cells compared with controls. Statistical significance was determined using the Mann–Whitney test (*p < 0.05; **p < 0.01).

To further characterize oxidative stress outcomes during ZIKV infection, we quantified major oxidative stress markers in infected HTR-8/SVneo cells. A significant increase in 8-hydroxy-2′-deoxyguanosine (8-OHdG), a well-established indicator of DNA damage, was observed in infected cells compared to controls (2.4 10^8^ ± 4.010^8^ × *vs*. 1.5 x10^8^ ± 2.0 ×10^8^ A.U.) (**Figures 7A, B**). In addition, the concentration of MDA, a byproduct of lipid peroxidation, was markedly elevated in infected cells relative to controls (0.01 ± 0.001 *vs*. 0.003 ± 0.002 nmol/mg protein) (**Figure 7C**), indicating potential damage to both DNA integrity and cellular membranes. Moreover, NO levels were significantly increased in infected cells compared to controls (95.68 ± 52.01 *vs*. 48.79 ± 9.18 μmol/ml) (**Figure 7D**), further supporting the presence of exacerbated oxidative stress and molecular damage during ZIKV infection.

## 4 Discussion

ZIKV infection in HTR-8/SVneo trophoblastic cells was standardized using a MOI 1 for 24 hours, yielding the highest proportion of infected and viable cells, as confirmed by NS1 detection through immunofluorescence and flow cytometry. At 48 hours post-infection, cell viability markedly declined, indicating progressive cytotoxicity, a phenomenon previously described in other cell types, including neural progenitors and macrophages (Tajik et al., 2024).

Importantly, the experiments were conducted using a Brazilian circulating ZIKV strain, ensuring that the findings reflect the pathogenic features of strains epidemiologically relevant to the national context. The PB81 isolate belongs to the Asian lineage associated with the 2015–2016 outbreak in the Americas and the emergence of Congenital Zika Syndrome (CZS). Epidemic Brazilian strains have been shown to efficiently infect neural progenitor cells and placental cell types, which may facilitate viral persistence and vertical transmission (Cugola et al., 2016; Tang et al., 2016). However, the development of CZS is multifactorial and depends not only on viral lineage but also on factors such as gestational timing of infection and host responses (Rasmussen et al., 2016).

Under physiological conditions, trophoblastic cells release microvesicles enriched with proteins, lipids, and nucleic acids, actively contributing to placental physiology (Tannetta et al., 2017). Electron microscopy revealed that ZIKV infection appears to enhance microvesicle formation and release, likely as a cellular response to viral stress, a pattern also observed in astrocytes, neurons, and macrophages (Holder et al., 2016; Huang et al., 2018; Zhou et al., 2019). While not the primary focus of this work, previous research (Holder et al., 2016) suggests that such vesicles may facilitate viral dissemination and modulate inflammatory responses in the host. Given the potential of these vesicles to act as long-range signaling mediators, further investigation into their role during Zika virus infections remains highly relevant to fully elucidate the mechanisms of viral pathogenesis and systemic spread.

Pronounced dilation of ER cisternae was also observed. This morphological alteration is commonly reported during flavivirus infections, including ZIKV, and reflects the extensive remodeling of the ER membrane required for the formation of viral replication complexes (Neufeldt et al., 2018; Cortese et al., 2020; Wolff et al., 2020b). The ER represents the primary replication hub for flaviviruses, including ZIKV (Cortese et al., 2017; Wolff et al., 2020a), where viral particles and replication complexes frequently accumulate (Mohd et al., 2020). Consistently, clusters of virus-like particles (VLPs) compatible with ZIKV were detected. ER membrane rearrangements may also promote functional interactions with other organelles, such as mitochondria, which are known to participate in metabolic and signaling processes required for efficient viral replication (Arakawa and Morita, 2019) ER remodeling, a hallmark of infection, creates microenvironments favorable for viral replication and hijacks host translation machinery to produce viral proteins (Tabata et al., 2016; Mohd et al., 2020).

Ultrastructural analysis also revealed loss of mitochondrial membrane integrity and cristae, indicating irreversible damage. Similar alterations were previously described by our group in placental tissues from infected patients (Rabelo et al., 2018) and by another group in animal models (Andrade et al., 2021). These ultrastructural changes, characterized by the loss of cristae and membrane integrity, are likely driven by the direct or indirect action of viral proteins on the mitochondrial architecture (Garcia-Blanco et al., 2016). Studies have shown that the ZIKV capsid (C) protein can associate with mitochondrial membranes, inducing oxidative stress and promoting mitochondrial fragmentation (Martins et al., 2024). Additionally, non-structural proteins, such as NS4B, have been implicated in the disruption of mitochondrial dynamics, leading to a decrease in mitochondrial membrane potential and subsequent metabolic failure (Han et al., 2021). Through the modulation of mitochondrial morphology and function, ZIKV may reprogram host cell metabolism to favor viral replication while simultaneously attenuating mitochondrial-dependent antiviral signaling pathways (Lee and Shin, 2023). Given the significant role of mitochondria in energy metabolism, immune signaling, apoptosis, and ROS generation (Marín et al., 2020), organelle dysfunction can severely compromise cellular homeostasis in the placental context (Marín et al., 2020).

Based on these morphological findings, Δψm was assessed, as it is essential for ATP production and oxidative phosphorylation control (Fisher et al., 2019) and is frequently used as a parameter of mitochondrial functionality. Infected cells exhibited a marked reduction in Δψm, consistent with the damage observed by electron microscopy. Similarly, García and colleagues (García et al., 2020) demonstrated that ZIKV-induced mitochondrial reorganization is accompanied by dissipation of the mitochondrial membrane potential, while Han and colleagues (Ishihara et al., 2003) showed that ZIKV promotes Δψm loss through Bax-mediated mitochondrial permeabilization, leading to activation of intrinsic apoptosis.

Because mitochondrial membrane potential plays a central role in regulating mitochondrial dynamics, the dissipation of Δψm prompted us to investigate whether genes controlling mitochondrial fission and fusion were also affected during ZIKV infection (Li et al., 2019). Mitochondrial fission is predominantly activated during organelle biogenesis or to segregate damaged mitochondria, regulated by proteins such as FIS1 and MFF, which recruit the cytosolic GTPase DRP1 (encoded by the *DNML1* gene) to the outer mitochondrial membrane (Zanfardino et al., 2025). Conversely, fusion is promoted under high energy demands and is mediated by MFN1 and MFN2 at the outer membrane and OPA1 at the inner membrane (Zanfardino et al., 2025). OPA1 is also essential for cristae maintenance, where respiratory chain complexes are localized (Barbier et al., 2017).

Gene expression analysis corroborated the observed mitochondrial damage. Genes related to mitochondrial fission (FIS1, MFF, *DNML1*) were downregulated post-infection. Although suppression of fission has been described in other flaviviral infections, such as dengue virus (DENV), where NS4B inhibits DRP1 and promotes mitochondrial elongation (Park et al., 2015; Yu et al., 2015), our findings indicate a broader dysregulation of mitochondrial dynamics. DRP1 inhibition may also facilitate NLRP3 inflammasome accumulation, caspase-1 activation, and IL-1β release, thereby contributing to exacerbated inflammatory signaling (Vásquez-Trincado et al., 2016).

Similarly, genes involved in mitochondrial fusion (MFN1, MFN2, OPA1) were significantly downregulated, reinforcing the disruption of mitochondrial dynamics. Dissipation of Δψm provides a mechanistic link between mitochondrial dysfunction and impaired fusion, as loss of membrane potential uncouples outer and inner membrane fusion machinery and activates the metalloprotease OMA1, which cleaves OPA1 and disrupts inner membrane organization (Yang et al., 2020), consistent with the ultrastructural damage observed. These findings align with previous reports demonstrating that ZIKV alters mitochondrial architecture in human neuronal models (Halling and Pilegaard, 2020) and other human cell systems (García et al., 2020).

Since mitochondrial dynamics and biogenesis are tightly interconnected processes that collectively maintain organelle homeostasis, we next evaluated the expression of PGC-1α, a master regulator of mitochondrial biogenesis (Li et al., 2019). In this context, we observed marked reductions in PGC-1α mRNA and protein levels. As a master regulator of mitochondrial metabolism, PGC-1α controls genes involved in mitochondrial enzyme synthesis, mitochondrial DNA replication, and antioxidant defense, including SOD, CAT, and GPx (Boytz et al., 2024). Its downregulation indicates impaired mitochondrial biogenesis and further supports the notion of compromised mitochondrial homeostasis.

Importantly, this coordinated transcriptional repression does not appear to represent a nonspecific consequence of viral infection. Although flaviviral infections are known to promote broad host transcriptional remodeling, including progressive suppression of multiple cellular pathways as replication increases (Bowen et al., 2017), transcriptional studies of ZIKV have consistently demonstrated selective gene expression programs characterized by upregulation of antiviral stress markers, inflammatory cytokines, and heat shock response genes during early infection (Chen et al., 2023). Therefore, the concomitant downregulation of genes involved in mitochondrial fusion, fission, and biogenesis suggests a specific disruption of mitochondrial homeostasis rather than a generalized shutdown of host transcriptional activity.

This selective suppression points toward a state of mitochondrial remodeling, a phenomenon increasingly recognized as a hallmark of flavivirus pathogenesis (Lee and Shin, 2023). In trophoblast models, the reduction in the expression of genes controlling mitochondrial dynamics may reflect a loss of organelle plasticity, which is essential for maintaining the metabolic demands of the placental barrier (Ma et al., 2018; Ledur et al., 2020). These findings in HTR-8/SVneo cells are consistent with observations in other placental cell lines, such as JEG-3, where ZIKV infection has been shown to alter mitochondrial morphology, and respiratory profile indicating that ZIKV manipulates mitochondrial homeostasis in placental cells (Ma et al., 2018; Chen et al., 2022; Lee and Shin, 2023). Viral proteins, including NS4A and NS4B, have been reported to localize to mitochondria and interfere with mitochondrial antiviral signaling protein (MAVS), thereby disrupting mitochondrial dynamics and innate immune signaling pathways (Yang et al., 2025). In addition, studies have demonstrated that ZIKV infection can promote mitochondrial fragmentation and impair membrane potential through the modulation of key regulators of mitochondrial fusion and fission, ultimately contributing to oxidative stress and cellular dysfunction (Halling and Pilegaard, 2020). Furthermore, the association between reduced mitochondrial gene expression, loss of membrane potential, and oxidative imbalance aligns with findings indicating that pathogen−induced mitochondrial dysfunction,characterized by impaired bioenergetics, altered dynamics, and excessive reactive oxygen species, contributes to disrupted host cellular homeostasis during infection (Vogel and Marcotte, 2012). Although direct mitophagic flux was not evaluated in this study, the convergence of transcriptional suppression, structural alterations, and oxidative imbalance supports a model in which ZIKV infection induces mitochondrial dysfunction, potentially contributing to the cytotoxic effects observed in gestational tissues.

Collectively, these findings indicate that ZIKV induces broad and multifactorial mitochondrial dysfunction, characterized by morphological damage, Δψm dissipation, impaired biogenesis, and disrupted organelle dynamics. Given that dysfunctional mitochondria represent one of major intracellular sources of reactive oxygen species (Wang et al., 2013), we next investigated whether the mitochondrial alterations observed during ZIKV infection were associated with redox imbalance and oxidative stress.

Under physiological conditions, cells possess efficient antioxidant systems, such as SOD and CAT, maintaining redox balance (Chen et al., 2023). SOD converts superoxide anions into hydrogen peroxide, whereas CAT converts hydrogen peroxide into water and oxygen (Bowen et al., 2017). However, ZIKV-infected HTR-8/SVneo cells exhibited a complex imbalance in enzyme expression and activity, suggesting that although infected cells may initially attempt to activate antioxidant defenses, these mechanisms become insufficient to counteract the excessive ROS production associated with mitochondrial dysfunction.

Despite reduced SOD mRNA, SOD protein levels (as seen by IF) and enzymatic activity were increased. This apparent discrepancy likely reflects post-transcriptional regulatory mechanisms frequently observed during cellular stress, where mRNA abundance does not necessarily correlate with protein levels or enzymatic activity. Large−scale proteomic and transcriptomic analyses demonstrate that mRNA levels often explain only part of the variance in steady−state protein abundance, with post−transcriptional, translational and protein degradation processes playing major roles (Cheng et al., 2016). Similarly, dynamic studies of stress responses highlight how protein changes can be decoupled from corresponding mRNA changes, further illustrating the role of post−transcriptional regulation (Sadi et al., 2014). In oxidative stress contexts, antioxidant enzymes can be rapidly regulated at the translational or post-translational level to counteract the accumulation of reactive oxygen species, allowing cells to mount an immediate defense independently of transcriptional activation. Such regulation may involve enhanced translation efficiency of stress-responsive transcripts or stabilization of antioxidant proteins through reduced proteasomal degradation (Vlasits et al., 2010; Van Ruyskensvelde et al., 2018). In the context of ZIKV infection, this response may represent an early adaptive attempt to neutralize the initial burst of superoxide anions generated by virus-induced mitochondrial dysfunction.

In contrast, CAT mRNA levels and enzymatic activity were both reduced, despite an apparent increase in CAT immunofluorescence intensity. This pattern may reflect oxidative inactivation of catalase rather than increased functional enzyme availability. Catalase is particularly susceptible to oxidative modifications of its catalytic heme group, which can impair enzymatic activity without immediately reducing protein abundance. Indeed, excessive hydrogen peroxide flux has been shown to inactivate catalase through oxidative damage, leading to the accumulation of structurally detectable but functionally compromised protein (Ma, 2013). Consistent with this interpretation, previous studies have reported that ZIKV infection decreases total catalase activity and promotes oxidative stress in infected cells, suggesting that the virus may actively modulate antioxidant pathways to maintain a pro-oxidant intracellular environment favorable for viral replication (Ma et al., 2018).

As a central transcriptional regulator of cellular antioxidant defenses, NRF2 controls the expression of multiple detoxifying and redox-balancing enzymes, including SOD and catalase, and its suppression can profoundly impair the cellular capacity to restore redox homeostasis (Almeida et al., 2020). The downregulation of NRF2 observed in our study may therefore contribute to the imbalance between antioxidant responses and ROS accumulation induced by ZIKV infection. These findings align with previous reports showing that ZIKV interferes with NRF2-dependent antioxidant pathways across diverse experimental models. For instance, Almeida et al (Wang et al., 2025). reported suppression of NRF2 signaling in human iPSC-derived astrocytes infected with ZIKV, while Ledur et al (Ma et al., 2018). observed impaired NRF2-mediated antioxidant responses and increased oxidative stress in infected murine tissues. Furthermore, ZIKV infection modulates antioxidant defenses in trophoblast-derived cell lines, including HTR-8/SVneo and JEG-3, resulting in oxidative stress and altered expression of redox-related genes (Ma et al., 2018; Halling and Pilegaard, 2020).

When antioxidant defenses fail to fully neutralize reactive species, oxidative damage to cellular macromolecules can occur. Elevated levels of 8-OHdG, a well-established marker of oxidative DNA damage, indicates that excessive ROS directly compromise genomic integrity, potentially impairing transcriptional processes and triggering apoptosis or autophagy (Del Rio et al., 2005). Concomitantly, increased MDA levels reflect enhanced lipid peroxidation, a process that destabilizes cellular membranes and generates secondary genotoxic products capable of inducing DNA mutations and tissue injury (Zhang et al., 2019).

In parallel, nitric oxide (NO) levels were increased. Although NO exerts protective signaling functions at physiological concentrations, its excessive production under oxidative conditions favors the formation of highly reactive nitrogen species, such as peroxynitrite, which amplifies lipid peroxidation, DNA fragmentation, and protein oxidation (Ma et al., 2018). Taken together, these alterations support a scenario in which ZIKV infection promotes oxidative stress, consistent with mitochondrial dysfunction and redox imbalance reported in previous studies (Sabouny et al., 2017; Ma et al., 2018; Vornic et al., 2024). In the placental context, excessive ROS production may overwhelm antioxidant defenses, contributing to adverse gestational outcomes such as preeclampsia and intrauterine growth restriction (Sekine and Youle, 2018).

In summary, ZIKV infection is associated with compromised mitochondrial function, as evidenced by transcriptional downregulation of dynamics-related genes, increased oxidative stress markers, and structural damage suggesting disrupted organelle quality control. These alterations create an intracellular environment conducive to cytotoxicity and viral persistence. Collectively, our findings enhance the understanding of ZIKV pathogenesis, specifically suggesting that this viral-induced mitochondrial dysfunction may be a critical contributor to adverse pregnancy outcomes. A deeper understanding of these mechanisms is thus essential for developing therapeutic strategies aimed at restoring mitochondrial integrity and mitigating the pathological effects of infection.

## Data Availability

The raw data supporting the conclusions of this article will be made available by the authors, without undue reservation.
